# Complete genome sequence of *Methylorubrum* sp. strain 263, a light-driven methylotrophic bacterium

**DOI:** 10.1128/mra.00912-26

**Published:** 2026-09-28

**Authors:** Miyu Morishita, Hideo Dohra, Nobuyuki Yoshida

**Affiliations:** 1 Department of Engineering, Graduate School of Integrated Science and Technology, Shizuoka University 13058, Hamamatsu, Shizuoka, Japan; 2 Shizuoka Instrumental Analysis Center, Shizuoka University 13058, Shizuoka, Japan; 3 Research Institute of Green Science and Technology, Shizuoka University 13058, Shizuoka, Japan; 4 Department of Science, Graduate School of Integrated Science and Technology, Shizuoka University 13058, Shizuoka, Japan; University of Rhode Island, Kingston, Rhode Island, USA

**Keywords:** methylotroph, light, oligotroph

## Abstract

*Methylorubrum* sp. strain 263 is a pink-pigmented facultative methylotrophic bacterium showing light dependence for methylotrophic growth. Here, we report the complete genome sequence of strain 263 consisting of a chromosome (5,298,893 bp) and a circular plasmid (44,302 bp).

## ANNOUNCEMENT

Recent studies have revealed that pink-pigmented facultative methylotrophs (PPFMs) live on the phyllosphere and exert growth-promoting effects on plants (1, 2). In our previous study, natural samples (e.g., soil and leaves) were cultivated in minimal liquid medium containing methanol under light irradiation. As a result, *Methylorubrum* sp. strain 263 was isolated from soil in Hamamatsu, Shizuoka, Japan (34°41′N, 137°38′E). This strain exhibited both oligotrophy and light-dependent methylotrophic growth (3). Complete genome sequencing of strain 263 is necessary to understand its unique light-dependent growth and accelerate the methanol-based bioeconomy.

Total DNA was extracted from 3 mL of the culture (3.14 × 10^9^ CFU/mL) of strain 263 with the medium containing 1% methanol as the sole carbon source (3) using NucleoBond HMW (Takara Bio, Shiga, Japan). For library preparation, 3 μg of genomic DNA was sheared using a Megaruptor 3 (Diagenode, Belgium) followed by size selection of approximately 7–12-kb fragments using AMPure PB magnetic beads (Pacific Biosciences, CA, USA). A sequencing library was prepared using the SMRTbell Prep Kit 3.0 and sequenced on the PacBio Revio platform using the Revio SPRQ chemistry with a 24-h movie time at Macrogen, Inc. (Seoul, South Korea). PacBio HiFi reads were filtered by retaining reads ≥5,000 bp using SeqKit v2.13.0 (4) with the “seq -m5000” option, resulting in 52,721 reads totaling 468,906,805 bp, with a read N50 of 9,379 bp and approximately 88-fold genome coverage. The filtered reads were *de novo* assembled using Flye v2.9.3 (5) with the --pacbio-hifi and --genome-size 5.4m options, yielding two circular contigs corresponding to a chromosome and a plasmid. The circular chromosome and plasmid sequences were rotated using Geneious Prime 2025.2 (6) to begin with the *dnaA* and *parA* genes, respectively. Gene prediction and annotation were performed using DFAST v1.3.7 (7) with an in-house reference database constructed from RefSeq genome records for the genus *Methylorubrum* downloaded in November 2025, followed by manual curation. Protein-coding, rRNA, and tRNA genes were predicted using GeneMarkS2 v1.14_1.25 (8), RNAmmer v1.2 (9), and tRNAscan-SE v2.0.5 (10), respectively. Default parameters were used, except where otherwise noted.

The genome of strain 263 consists of a circular chromosome (5,298,893 bp, 69% guanine + cytosine [G + C]) and a circular plasmid (44,302 bp, 66% G + C). The strain 263 chromosome has 4,869 coding sequences (CDSs), 5 sets of rRNA genes, and 57 tRNA genes. The average nucleotide identity (ANI) values between the genome sequence of strain 263 and those of 11 species of the genus *Methylorubrum* were calculated using calcANI.pl (https://github.com/Computational-conSequences/SequenceTools) based on the legacy blast algorithm (11). Strain 263 showed the highest ANI value (90.37%) with *Methylorubrum podarium* EM12 (12), suggesting that strain 263 represents a novel species within the genus *Methylorubrum*.

## Data Availability

The nucleotide sequences of strain 263 have been deposited in the DDBJ/EMBL/GenBank databases under accession numbers AP051725 (chromosome) and AP051726 (plasmid pMM263). The raw data are available under DRA accession number DRR1070462. All project data are available under BioProject accession number PRJDB42666.
